# Draft Genome Sequence of a Biocontrol Rhizobacterium, *Chryseobacterium kwangjuense* Strain KJ1R5, Isolated from Pepper (*Capsicum annuum*)

**DOI:** 10.1128/genomeA.00301-16

**Published:** 2016-04-21

**Authors:** Jin-Ju Jeong, Hongjae Park, Byeong Hyeok Park, Mohamed Mannaa, Mee Kyung Sang, In-Geol Choi, Ki Deok Kim

**Affiliations:** a Laboratory of Plant Disease and Biocontrol, College of Life Sciences and Biotechnology, Korea University, Seoul, Republic of Korea; b Department of Biotechnology, Korea University, Seoul, Republic of Korea; c Division of Agricultural Microbiology, National Academy of Agricultural Science, Rural Development Administration, Jeonju, Republic of Korea

## Abstract

Strain KJ1R5 of the rhizobacterium *Chryseobacterium kwangjuense* is an effective biocontrol agent against Phytophthora blight of pepper caused by a destructive soilborne oomycete, *Phytophthora capsici*. Here, we present the draft genome sequence of strain KJ1R5, which contains genes related to biocontrol, plant growth promotion, and environmental stress adaptation.

## GENOME ANNOUNCEMENT

Biological control of plant diseases with various microorganisms is considered an attractive, environmentally sound alternative to agricultural chemicals, which can cause environmental hazards, crop toxicity, or the development of chemical-resistant pathogenic strains (1). A number of antagonistic bacteria derived from plant rhizospheres have potential applications as biocontrol and/or plant growth-promoting agents (2, 3). Among the limited number of biocontrol species in the genus *Chryseobacterium* (4, 5), *Chryseobacterium kwangjuense* strain KJ1R5, examined in this study, is an effective biocontrol rhizobacterium against Phytophthora blight of pepper caused by the soilborne oomycete *Phytophthora capsici* under controlled and field conditions (6, 7). This biocontrol strain might also reduce ginseng root rot caused by *Phytophthora cactorum* (8). The biocontrol activity of the strain might be related to its ability to colonize the rhizosphere of plant roots (6). The yellow-pigmented, Gram-negative, and rod-shaped *C. kwangjuense* KJ1R5, which was reported as a novel *Chryseobacterium* species in our previous study (9), was originally isolated from the rhizoplane of the pepper root in a field (Kwangju, South Korea) in 2001.

The genome of strain KJ1R5 was sequenced using the Illumina MiSeq platform at the Computational and Synthetic Biology Laboratory, Department of Biotechnology, Korea University (Seoul, South Korea). We obtained 1,387,560 reads (832.5 Mb) from the paired-end sequencing of a genomic library, with an average insert size of 500 bp. We trimmed the low-quality reads with a quality threshold of Q30. The filtered reads were *de novo* assembled using the SPAdes assembler (10); the resultant assembly consisted of 22 scaffolds with a total length of 5,087,431 bp (163.6-fold coverage) and a G+C content of 38.49%. The maximum scaffold length was 2,133,668 bp, and the *N*_50_ of the assembly scaffolds was 1,120,681 bp. The annotation was performed with the NCBI Prokaryotic Genome Annotation Pipeline (PGAP) service. In total, 4,445 coding sequences were predicted by PGAP, 4,096 (92.2%) of which showed sequence similarity to known genes in the NCBI database. We retrieved 66 tRNA, one 16S rRNA, one 23S rRNA, and six 5S rRNA sequences.

The genome analysis of strain KJ1R5 revealed genes associated with biocontrol activity, such as antimicrobial activity-related genes (e.g., those involved in polyketide and proteinase synthesis and the processing or transport of antibiotics) (11) and genes with functions related to plant growth promotion, such as ammonia and siderophore production, pyrroloquinoline quinone, and phosphate solubilization (12–14). The analysis also revealed genes that encode antioxidant enzymes, such as superoxide dismutase, catalase, and hydrogen peroxidase, which are responsible for removing free radicals and preventing cell damage for abiotic or biotic stress management (15). In conclusion, the genome of *C. kwangjuense* KJ1R5 will enable an in-depth understanding of the biocontrol activity, plant growth promotion, and environmental stress adaptation of the strain.

### Nucleotide sequence accession numbers.

This whole-genome shotgun project has been deposited in the DDBJ/EMBL/GenBank under the accession no. LPUR00000000. The version described in this paper is the first version, LPUR01000000.
